# Educational technologies to guide pulmonary tuberculosis sputum collection: a systematic review^*^

**DOI:** 10.1590/1980-220X-REEUSP-2021-0433en

**Published:** 2022-07-22

**Authors:** Karine Nascimento da Silva, Paula Suene Pereira dos Santos, Rayanne de Sousa Barbosa, Maria do Socorro Vieira Lopes, Antonio Germane Alves Pinto, Edilma Gomes Rocha Cavalcante

**Affiliations:** 1 Universidade Regional do Cariri, Programa de Pós-Graduação em Enfermagem, Crato, CE, Brazil.

**Keywords:** Tuberculosis, Pulmonary, Educational Technology, Health Education, Sputum, Systematic Review, Tuberculosis Pulmonar, Tecnología Educacional, Educación en Salud, Esputo, Revisión Sistemática, Tuberculose Pulmonar, Tecnologia Educacional, Educação em Saúde, Escarro, Revisão Sistemática

## Abstract

**Objective:**

To evaluate the contributions of educational technologies used during the guidelines for sputum collection from pulmonary tuberculosis.

**Method:**

Systematic review guided by Preferred Reporting items for Systematic Reviews with protocol registered in the database *International Prospective Register of Systematic Reviews*, with number CRD42020208162. The search was performed in the Cinahl, Scopus, PubMed, Web of Science, Embase, Lilacs, CENTRAL, CAPES, Proquest, OpenGrey databases and manual search in the reference list. The search, selection of studies, data extraction, and methodological evaluation using the Cochrane Risk-of-Bias tool were performed by two independent reviewers.

**Results:**

A total of 2,488 studies were evidenced, with seven being selected and analyzed, of which four used structured lectures; three, educational booklet; and one, an educational video, used alone or together, impacting the bacteriological confirmation of tuberculosis. The studies had a low risk of bias.

**Conclusion:**

Scientific evidence has shown that educational technologies contribute to increasing the quality, volume, and appearance of the sputum sample, which improves the bacteriological confirmation of the disease.

## RESUMO

**Objetivo:**

Avaliar as contribuições das tecnologias educativas utilizadas durante as orientações para coleta de escarro da tuberculose pulmonar.

**Método:**

Revisão sistemática guiada pelo *Preferred Reporting items for Systematic Reviews* com protocolo registrado na base *International Prospective Register of Systematic Reviews*, sob o número CRD42020208162. A busca foi realizada nas bases de dados Cinahl, Scopus, PubMed, Web of Science, Embase, Lilacs, CENTRAL, CAPES, Proquest, OpenGrey e busca manual na lista de referências. A busca, seleção dos estudos, extração dos dados e avaliação metodológica pela ferramenta do Risco de Viés da Cochrane foram realizadas por dois revisores independentes.

**Resultados:**

Evidenciaram-se 2488 estudos, sendo sete selecionados e analisados, dos quais quatro utilizaram palestras estruturadas; três, *folder* educativo; e um, vídeo educativo. Todos foram utilizados de forma isolada ou em conjunto, impactando na confirmação bacteriológica da tuberculose. Os estudos apresentaram baixo risco de viés.

**Conclusão:**

As evidências científicas demonstraram que as tecnologias educativas apresentam como contribuições o aumento da qualidade, volume e aspecto da amostra de escarro, o que proporciona a melhoria da confirmação bacteriológica da doença.

## RESUMEN

**Objetivo:**

Evaluar las contribuciones de las tecnologías educativas utilizadas durante las orientaciones para colecta de esputo de la tuberculosis pulmonar.

**Método:**

Revisión sistemática guiada por el Preferred Reporting items for Systematic Reviews con protocolo registrado en la base *International Prospective Register of Systematic Reviews*, bajo el número CRD42020208162. La búsqueda fue realizada en las bases de datos Cinahl, Scopus, PubMed, Web of Science, Embase, Lilacs, CENTRAL, CAPES, Proquest, OpenGrey y búsqueda manual en la lista de referencias. La búsqueda, selección de los estudios, extracción de los datos y evaluación metodológica por la herramienta del Riesgo de Sesgo Cochrane fueron realizadas por dos revisores independientes.

**Resultados:**

Se evidenciaron 2488 estudios, siendo siete seleccionados y analizados, de los cuales cuatro utilizaron palestras estructuradas, tres *folder* educativo y un vídeo educativo, utilizados de forma aislada o conjunta, impactando en la confirmación bacteriológica de la tuberculosis. Los estudios presentaron bajo riesgo de sesgo.

**Conclusión:**

Las evidencias científicas demostraron que las tecnologías educativas presentan como contribuciones el aumento de la calidad, volumen y aspecto de la muestra de esputo, lo que proporciona el perfeccionamiento de la confirmación bacteriológica de la enfermedad.

## INTRODUCTION

Pulmonary tuberculosis (TB) is the main way to maintain disease transmission in bacilliferous people, and for its control the search and investigation of respiratory symptomatic people (RS) are required^(1)^. The classification of the RS person as a criterion for the investigation of pulmonary TB presents good accuracy^(2)^.

The discontinuity of care and the inexistence of segments related to the results of sputum smears lead to a reduction in the number of RS people investigated, resulting in a consequent decrease in confirmed cases and an increase in the transmissibility of the disease^(3)^. It is known that bacilloscopy has a sensitivity of 55.1% and a specificity of 99.6%^(4)^, presenting diagnostic relevance, as well as being a simple, safe, and low-cost method^(5)^.

It should be noted that low bacilloscopy sensitivity refers to the need for 5,000 to 10,000 bacilli per ml for a positive test^(6)^. Thus, to produce a sample with the recommended quantity, quality, and appearance, health education, exemplifying the importance of carrying out the collection according to the guidelines, should be prioritized^(7–8)^.

It is also noteworthy that the use of educational technologies during the provision of guidelines improves the effectiveness of the diagnosis and reduces the false negatives, which demonstrates great potential to reduce the chain of transmission of TB. Therefore, the conveyance of the recommended guidelines for sputum collection provides improvements in the diagnostic capacity of health services, helping to control the disease^(9)^.

Guidance on the steps of sputum collection associated with the use of educational technologies facilitates replication by RS people. These provide knowledge of the steps for sputum collection, facilitate understanding of the importance of diagnosis, disease monitoring, and subsequent treatment effectiveness^(10–11)^.

In this regard, access to information on the importance of these guidelines leads to an improvement in sputum quality, demonstrating that the diagnosis of TB does not necessarily require the introduction or production of new diagnostic methods. A focus on sputum quality significantly increases bacteriological confirmation and should be incorporated to improve the sensitivity and accuracy of TB diagnosis^(12)^.

The development of a systematic review on the contributions of educational technologies for sputum collection proves to be relevant in supporting the implementation of educational technologies in clinical practice, as a resource to assist in the collection of sputum with quantity, quality, and recommended aspects, helping for bacteriological confirmation of pulmonary TB. Furthermore, it should be noted that searching did not find any ongoing, completed, or published research protocol in the Prospective International Registry of Systematic Reviews (PROSPERO), in the Cochrane Database of Systematic Reviews, or in PubMed.

Thus, given the knowledge gap in the use of educational technologies that point to scientific evidence regarding sputum collection from pulmonary TB and support the professional practice regarding these guidelines for RS people, the planning, design, and publication of the registration with PROSPERO to promote transparency and avoid duplication of efforts were started. Thus, we sought to investigate the following research question: What evidence is available in the scientific literature regarding the contributions of the use of educational technologies at the time of guidance for pulmonary tuberculosis sputum collection?”.

Therefore, the objective was to evaluate the contributions of educational technologies used during the guidelines for sputum collection from pulmonary tuberculosis.

## METHOD

### Design of Study

This is a systematic literature review, developed according to the criteria of the *Preferred Reporting Items for Systematic Reviews and Meta-Analyses* (PRISMA)^(13)^, with PROSPERO registered protocol of number CRD42020208162. The study used secondary data, and approval by the Research Ethics Committee was not required.

The PICO strategy (P – *population*; I – *intervention*; C – *comparison*; O – *outcomes*) guided the elaboration of the guiding question: “What evidence is available in the scientific literature regarding the contributions of the use of educational technologies at the time of guidance for pulmonary tuberculosis sputum collection?”.

The eligibility criteria for the selection of articles were RS people for pulmonary TB, with minimum age of 18 years, or health professionals who carried out the guidelines for sputum collection (population); use of educational technologies (types of educational technologies used: booklets, flipcharts, brochure, educational videos, and messages) during the guidelines for pulmonary tuberculosis sputum collection (intervention); comparison of the usual guidance without the use of educational technologies as an ancillary method (comparison); contributions of use in terms of improving appearance, quality, sample quantity and bacteriological confirmation (outcomes).

It should be noted that the comparator was considered, but did not constitute a requirement for inclusion in the research. There were no restrictions on the language or year of publication of the studies analyzed to expand the findings. Duplicate or repeated studies were excluded.

### Search Strategy

The search was carried out in October 2020 in the databases Cumulative Index to Nursing and Allied Health Literature (CINAHL), Scopus, PUBMED, Latin American and Caribbean Literature in Health Sciences (Lilacs), Web of Science (WoS), Embase and Cochrane Central Register of Controlled Trials (CENTRAL). The gray literature sources were accessed through the Catalog of Theses and Dissertations of the Coordination for the Improvement of Higher Education Personnel (CAPES), from the *ProQuest* (Dissertations & theses) and *OpenGrey*. Also, a manual search of references of the articles selected for full reading was carried out.

**Chart 1.**
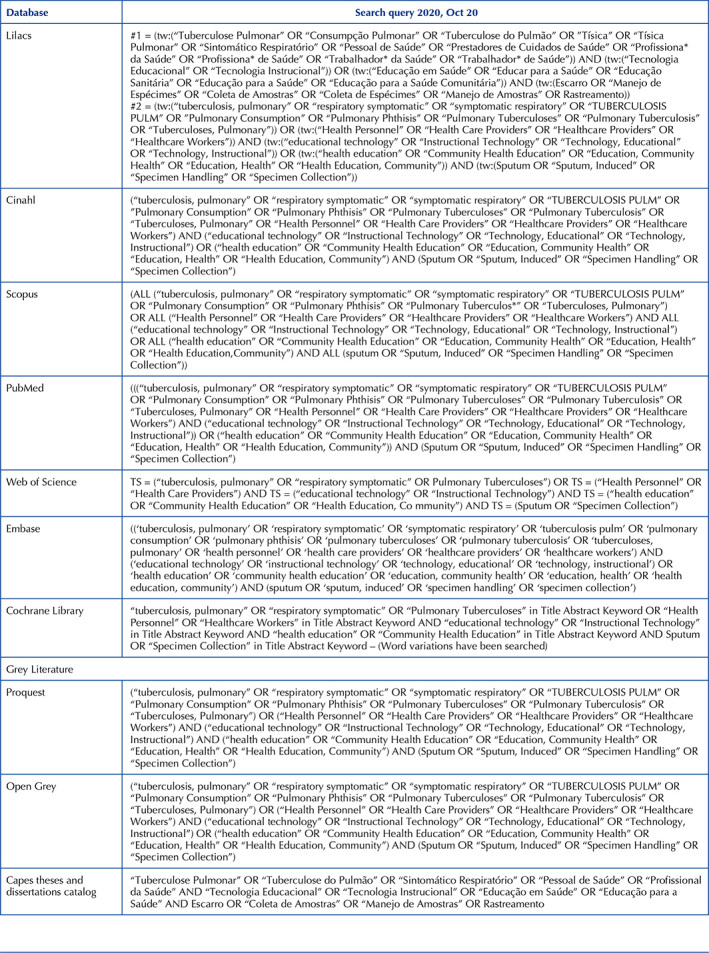
Search strategy in the consulted databases – Crato, CE, Brasil, 2021.

Controlled terms were extracted from the Health Sciences Descriptors (DECS) and the Medical Subject Headings (MeSH). These were combined using the Boolean operators OR and AND. The search strategy was adapted according to the electronic databases, with search terms in all fields. It should be noted that in LILACS, which is a database with articles in Portuguese, the search was also carried out with the DECS descriptors: “Pulmonary Tuberculosis”, “Health Personnel”, “Educational Technology”, “Health Education”, Sputum and “Specimen Handling”, and the respective synonyms, as shown in Chart 1.

### Data Selection and Extraction

The review was carried out by three reviewers, one of them being an expert on the theme worked on, with the help of two experts in systematic review studies for planning development, research question formulation, search strategy, and protocol registration.

Initially, the search in the databases, the screening of findings, data selection and extraction were performed by two independent reviewers simultaneously.

It was established at the time of planning that, in case of disagreement between the two reviewers, the third reviewer, with expertise in the theme worked, would be requested for the final decision. Although the research had the third reviewer, there were no disagreements. It should also be noted that initial tests were carried out to adapt the collection process. Data selection and extraction were carried out between November 2020 and February 2021. For this stage of initial reading and selection of articles, the reference manager instrument, *Rayyan Qatar Computing Research Institute*, was used (https://rayyan.ai/users/sign_in).

The initial selection was performed through the analysis of the title and abstract. Data were collected using a specific form, based on the following information: article identification (author(s), title, journal/database, country, study language and year of publication); goals; methodological approach (design, participant characteristics, sample size, loss to follow-up, inclusion and exclusion criteria, collection, intervention and control characteristics, confounding factors, outcome characteristics, data organization and analysis); description of educational technologies (type, application, duration, evaluation, professional who applied the technology, facilities, difficulties, benefits and contributions); main results and changes in results, including changes in sample appearance, quality, quantity, and bacteriological confirmation; and conclusions.

### Methodological Evaluation

The Classification of the Level of Evidence (LE) was carried out in accordance with what was proposed by the *Oxford Center for Evidence-Based Medicine*, in an adapted version^(14)^, defined in: 1A – systematic review (with homogeneity) of controlled and randomized clinical trials; 1B – controlled and randomized clinical trial with narrow confidence interval; 1C – therapeutic results; 2A – systematic review (with homogeneity) of cohort studies; 2B – cohort study (including lower quality clinical trial); 2C – observational study of therapeutic results (*outcomes research*); 3A – systematic review (with homogeneity) of case- control studies; 3B – case-control study; 4 – case report (including cohort or lower quality case-control); and 5 – opinion without critical evaluation, based on consensus, physiological studies, with biological materials or animal models.

### Data Analysis and Treatment

Risk-of-bias assessment was carried out by two researchers, using the tool *RevMan*, based on the recommendations *Cochrane from the Cochrane Collaboration Handbook* for Systematic Reviews of Interventions, version 5.1.0. Critical tool for evaluating the reliability, relevance, and results of published studies, in which seven domains were evaluated: I) Allocation of the randomization sequence (selection bias); II) Allocation concealment (selection bias); III) Blinding of the participants and the team involved (performance bias); IV) Blinding of outcome assessors (detection bias); V) Incomplete outcomes (attrition bias); VI) Selective outcome report (publication bias); and VII) Other sources of bias^(15)^.

The evaluated outcome was related to the contributions of educational technologies in sputum collection in terms of improving the collected sputum appearance, quantity, and quality, being considered as an outcome measure.

Due to the methodological differences of the analyzed studies, the quantitative summarization of the results through the meta-analysis became unfeasible. Thus, the meta-analysis was not performed because it did not have enough data regarding similarities in the types of educational technologies and methodological design^(15)^, with a comparison between them not being made, proceeding with the descriptive analysis of the evidenced results.

## RESULTS

### Selection of Studies

**Figure 1.**
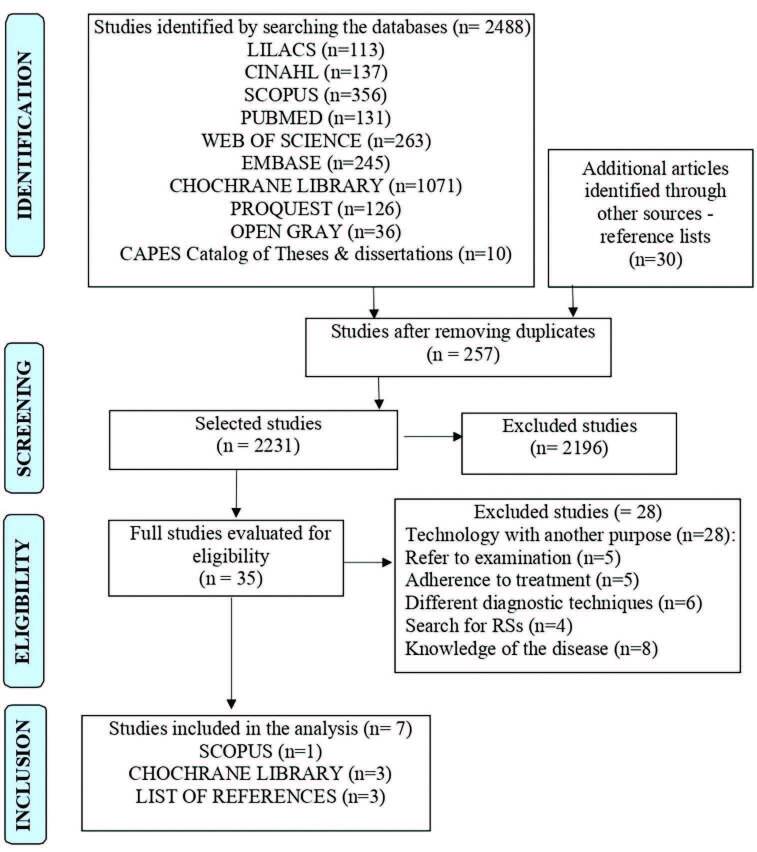
Selection flowchart for the Systematic Review articles. Crato, CE, Brazil, 2021.

Of the 2,488 articles identified in the 10 electronic databases and in the manual search, 257 were removed because they were duplicates and 2196 were selected for titles and abstract reading. Of these, 35 studies were read in full. After this exhaustive phase, seven studies were selected, and included for the final analysis of the qualitative synthesis, according to Figure 1.

### Characterization of the Studies

As for the characteristics of the studies, the publication date of the seven articles included ranged between 2005 and 2019. All were published in English, with six studies with a randomized controlled clinical trial design and one quasi-experimental, conducted in different countries. A total of 2050 people participated in the studies. Of these, 2638 used educational technologies in the interventions and 2605 received unstructured guidelines and without the use of educational technologies in the control groups. Among the educational technologies, structured lectures were used (n = 4)^(9,12,16–17)^, educational brochure (n = 3)^(9,18–19)^, instructional video (n = 1)^(20)^ and sputum induction if necessary (n = 2)^(12,17)^, associated with another educational technology (n = 1)^(12)^.

**Chart 2.**
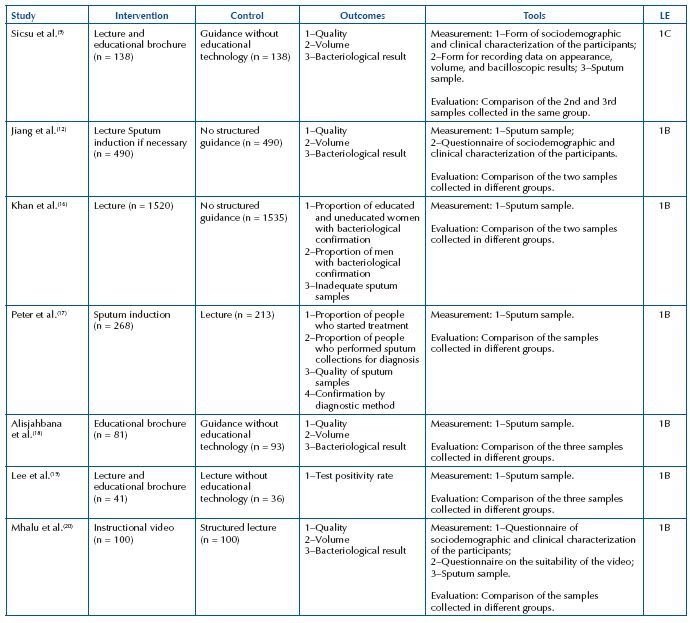
Characterization of the studies regarding the applied intervention – Crato, CE, Brazil, 2021.

With regard to outcomes, most studies addressed quality, volume and bacteriological results (n = 4)^(9,12,18,20)^. In the measurement tools, the sputum sample was used in all studies, followed by the sociodemographic and clinical characterization questionnaire of the participants (n = 3)^(9,12,20)^. As for the evaluation, it was evidenced that all studies compared sputum samples, as shown in Chart 2.

*LE – Level of Evidence

### Characterization of Technologies

In the characterization of the technologies used, the type of technology, choice criteria, form of applicability, duration of use, professional who applied it and the facilities were identified. Oral technology was the most used (n = 5)^(9,12,16–17,19)^, followed by printed (n = 3)^(9,18–19)^ and digital (n = 1)^(20)^.

Among the selection criteria, it was observed that the criterion for oral technology varied between simplicity and^(12)^ utility for female audience^(16)^ and strategy for identifying whether instruction or induction would be more effective^(17)^. Thus, the simplicity of replication was demonstrated as facilities of oral technologies^(12,16)^, the low cost with the possibility of being used in countries that have fewer resources^(16)^ and to use it in association with other educational technology^(12)^.

Regarding the form of applicability, all interventions were applied individually, in a specific room dedicated to the reception of the RS person who would receive guidance from a trained professional using educational technology. Variations were observed depending on the type of technology used, and in all of them the recommended steps for sputum collection were demonstrated. The duration of use ranged from 2^(16)^ and 20 minutes^(9)^. The professional who applied the technology was mostly nurses (n = 2)^(9,12)^.

Regarding the main contributions identified with the use of educational technologies, it was found that most educational technologies provided an improvement in the increase in the volume and quality of the sputum collected, which leads to the bacteriological confirmation of pulmonary TB (n = 5)^(9,12,16,18,20)^, consequently improved the yield and efficiency of bacteriological examinations.

Furthermore, studies (n = 2)^(17,19)^ that did not verify contributions of these technologies were identified, such as the use of educational brochure^(19)^ which did not result in an increase in the positivity of the exam when compared to the group that obtained the structured lecture. It should be noted that this study defined the test’s positivity rate as the assessment outcome, not considering criteria such as quality and volume of the sputum sample.

**Chart 3:**
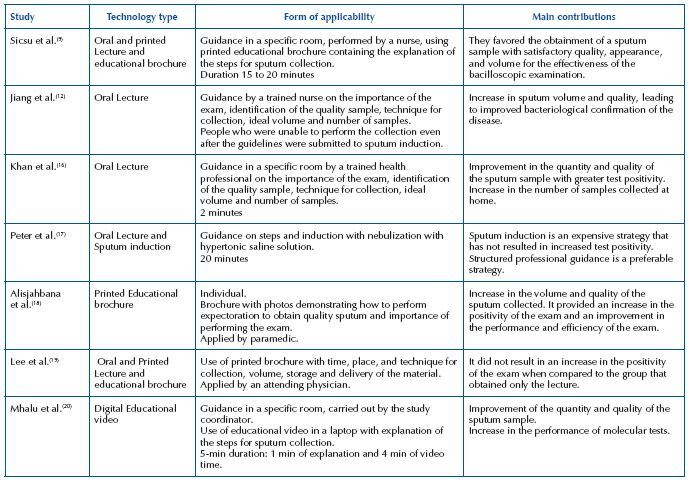
Characteristics of the technologies identified in the selected studies – Crato, CE, Brasil, 2021.

In another study^(17)^, sputum induction was considered an expensive strategy that did not result in greater test positivity. Thus, both studies^(17,19)^ demonstrated that the structured lecture, defined as an educational technology, would be more appropriate, according to Chart 3.

### Internal Validity Of Included Studies

**Figure 2.**
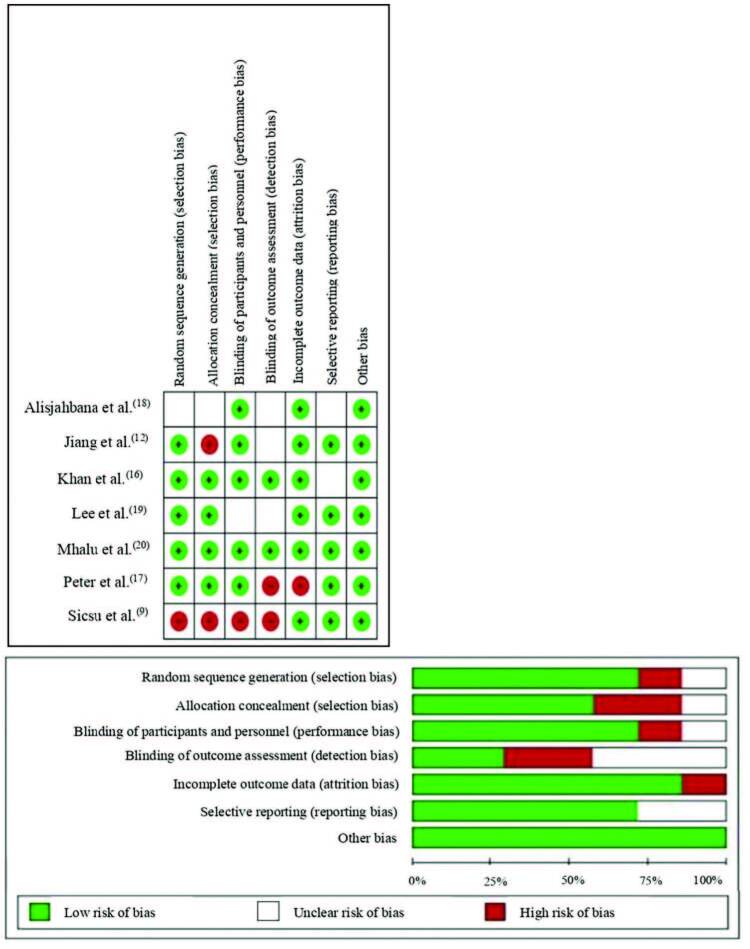
Risk of bias of the studies included and evaluated by the Cochrane Collaboration tool. Crato, CE, Brasil, 2021.

Regarding the risk of bias of the studies, it was identified that, in most studies, there was a low risk of selection bias regarding the generation of random sequence (71%) and allocation concealment (57%). Regarding performance bias and attrition bias (71%), there was a low risk of bias. Regarding detection bias, the majority (43%) were classified as having an uncertain risk of bias, as shown in Figure 2.

## DISCUSSION

The present study evidenced the educational technologies at the moment of guidance for the collection of sputum of pulmonary TB and its contributions, with the most used being the lecture, the brochure and the educational video. Regarding the methodological quality of the studies, it was identified that most studies had a low risk of bias.

As for the investigated outcomes, it is necessary to evaluate not only the results obtained in the exams, but other things, such as most of the studies included in this review that evaluated the quality and volume of the samples collected. In fact, a more comprehensive assessment can demonstrate the contributions of educational technologies more accurately, when considering the essential variables for the bacteriological result of the tests^(21)^. Thus, the importance of evaluating such outcomes is evident for a better understanding of the effect of the educational technologies used.

Moreover, in this study, it was noticed that, in most of the researches, the control group was defined according to the accomplishment of the guidance without the use of educational technologies^(9,18–20)^ or unstructured information^(12,16)^. These controls were used to compare the usual practices of guidance in health services with the use of simple and low-cost educational technologies.

In this review, five studies indicated the use of structured lectures^(9,12,16–17,19)^, with variations in its use being observed. In most studies, the lecture was applied in combination with other strategies, such as sputum induction^(12,17)^ and educational brochure^(9,19)^ or individual use^(16)^. Among these, all presented significant contributions regarding the improvement of the characteristics of the sputum collected, evidenced in the volume, quality and bacteriological results.

Thus, it is understood that health education organized in a structured way, through lectures, provides a significant improvement in knowledge with an impact on the behavior of RS people with pulmonary TB. For this, variables capable of raising the level of understanding shall be considered, such as the appropriate time for each session, content covered, and its association with printed or digital materials to provide appropriate motivation and facilitate the learning of the content covered^(22)^.

It is noticed that, in the context of health services, in general, the guidelines provided to SR people with TB on sputum collection happen in a punctual, unstructured way, with no information including the necessary steps to acquire a sputum sample of good quality or the use of educational technologies.

In this review, three studies were identified that used the educational brochure^(9,18–19)^, being used alone^(18)^ and associated with the lecture^(9,19)^. As for the contributions, in most of them, there was an increase in the numer of sputum samples of better quality, satisfactory appearance and volume for the effectiveness of bacteriological tests.

In addition to counseling, the use of additional strategies such as leaflets allows patients to understand and put the guidelines provided into practice, especially because of the possibility of reading the information again whenever they wish, strengthening knowledge about the disease and its complications^(23)^. In fact, implementation according to local resources and the needs of RS people with TB facilitates adherence to the recommendations^(24)^.

It was identified that the use of educational video^(20)^, applied individually as a strategy, improved the quantity and quality of the sputum sample, increasing the performance of bacteriological tests.

The increased access to mobile devices allows the use of educational videos in situations with access barriers, especially for people with a lower level of literacy, making it effective in improving general knowledge about TB prevention, diagnosis, and treatment. This way, digital technologies should be part of health education strategies by TB control programs, made available in open access and free of charge, due to their potential to reduce the incidence of the disease. This way, the educational video proves to be a powerful, low-cost, sustainable technology that improves access to knowledge about TB^(11)^.

Through the analysis of the characteristics of the technologies, the use of printed, digital and oral technologies was verified. The applicability took place individually, quickly, and in a specific room for guidance of the RS people by a trained professional, mostly nurses. Given the above, it is evident that the necessary resources for implementation are already present in health services, including those with less financial resources. In practice, the professionals who deliver the materials needed for collection, such as the collection pot, also guide on the steps and must use such educational technologies to facilitate the understanding of the information available on sputum collection.

It was evidenced, in this study, that the interventions applied during the guidelines for the collection of quality sputum varied among educational brochures, structured lectures, and instructional videos. The selection criteria ranged from simplicity, usefulness to the target audience, ease of application, low cost, and the possibility of using them in an associated way. This structured counseling by trained professionals leads to the improvement of detailed knowledge of all important aspects of TB, characterizing itself as a necessary intervention for the diagnosis of the disease^(25)^.

Thus, this synthesis of scientific evidence can help to implement technologies in the health services, such as educational brochures, lectures, and instructional videos, for the provision of guidelines in health services, as resources to assist in the guidance for sputum collection in pulmonary TB.

With regard to limitations, it was found that it was impossible to carry out a meta-analysis of the data, due to the significant heterogeneity of the included studies. Thus, further studies, with representative samples and rigorous methodological designs, are required to confirm these findings, since some variables were evaluated with an uncertain risk of bias.

## CONCLUSION

The evidence from this review demonstrates a low risk of bias. The educational technologies used for guidance on the steps of sputum collection were structured lectures, brochures, and educational video, which were applied alone or in association.

Among the main contributions, it became evident that educational technologies, by facilitating access to information on the recommended steps for acquiring a good quality sputum sample, provide an increase in quality, volume and bacteriological appearance, leading to improved confirmation of the disease and with the potential to increase the diagnostic capacity of these tests.

This way, educational technologies are shown to be tools that help the work and dialogue of nurses in health education with the RS person and family in view of the need for sputum collection. Thus, the daily use of such technologies facilitates and standardizes the guidelines related to the necessary steps to acquire sputum with the aspect, quantity, and quality recommended for bacteriological confirmation of the disease, with the potential to increase the diagnostic capacity for pulmonary TB.
